# Computational-Model-Based Analysis of Context Effects on Harmonic Expectancy

**DOI:** 10.1371/journal.pone.0151374

**Published:** 2016-03-22

**Authors:** Satoshi Morimoto, Gerard B. Remijn, Yoshitaka Nakajima

**Affiliations:** 1 Department of Design, Graduate School of Design, Kyushu University, Fukuoka, Japan; 2 Research Center for Applied Perceptual Science, Kyushu University, Fukuoka, Japan; 3 Department of Human Science, Faculty of Design, Kyushu University, Fukuoka, Japan; University of Salamanca- Institute for Neuroscience of Castille and Leon and Medical School, SPAIN

## Abstract

Expectancy for an upcoming musical chord, harmonic expectancy, is supposedly based on automatic activation of tonal knowledge. Since previous studies implicitly relied on interpretations based on Western music theory, the underlying computational processes involved in harmonic expectancy and how it relates to tonality need further clarification. In particular, short chord sequences which cannot lead to unique keys are difficult to interpret in music theory. In this study, we examined effects of preceding chords on harmonic expectancy from a computational perspective, using stochastic modeling. We conducted a behavioral experiment, in which participants listened to short chord sequences and evaluated the subjective relatedness of the last chord to the preceding ones. Based on these judgments, we built stochastic models of the computational process underlying harmonic expectancy. Following this, we compared the explanatory power of the models. Our results imply that, even when listening to short chord sequences, internally constructed and updated tonal assumptions determine the expectancy of the upcoming chord.

## Introduction

When one listens to music, expectancy for a following musical event is formed internally based on the preceding context [1–3]. Particularly in a tonal music, as represented by Western classical music, patterns of expectancy are actively utilized to build a flow of harmonic movement to a center chord. In Western classical music theory, the center chord is called the tonic, and the patterns of expectancy are arranged as a practical guide to establish a sense of stability toward the tonic. For example, the “dominant” chord is considered to evoke a strong expectancy of an upcoming, more stable chord, so that a chord progression to the tonic is expected.

Musical expectancy is thought to involve emotion or mood induction (for a review, see [4]), and is studied in psychology through behavioral experiments [5]. Especially with regard to expectancy for an upcoming chord in a chord progression, i.e., harmonic expectancy, many studies have attempted to explain the influence of preceding musical events [6–16]. These studies suggested two main effects on harmonic expectancy: the local context effect and the global context effect [8]. The local context effect is formed internally through musical experience [9], and is built on the relationship between two successive chords [10]. The global context effect also seems to be formed through musical experience [11], and is related to the tonal context of the preceding events [8, 12]. To simulate the mechanism behind the global context effect, a spread activation model has been proposed [17]. In this model, activation caused by a played chord continues to spread through the network until it reaches an equilibrium state. This model can explain the automatic and speedy build-ups of related keys [18] and the robustness of temporal order in chord sequences [19].

It has to be noted that these previous studies implicitly assumed an agreement between the rules in Western music theory and corresponding perceptual tendencies. For example, in the spread activation model [17], each key connected with chords that were in that key. This seemed to be valid from the viewpoint of Western music theory. However, Western music theory is basically a practical rule set to compose a tonal music, and there has been no evidence that listeners have internal representations of keys and chords associated with them [20].

The purpose of the present study was to illustrate how harmonic expectancy arises from observation of chord sequences. In order to simulate the underlying mechanism, we proposed a stochastic modeling method: We modeled in various ways how participants internally processed chord sequences, and compared performances of these models. This type of approach, a computational-model-based analysis, has been developed in the field of computational neuroscience to explain and estimate cortical hemodynamic responses, especially for complex cognitive processes like learning and decision making (see [21, 22], for a review). Stochastic models are also widely used in sound processing. For example, hidden Markov models (HMMs) are practically used for modeling time-varying sequences, particularly for speech recognition [23]. In music informatics, HMMs have been utilized for finding keys in musical sequences [24, 25]. Assuming that humans assign a similar stochastic framework to musical contexts, stochastic modeling can be an effective tool to analyze harmonic expectancy without referring to Western music theory.

Participants performed a chord-sequence-rating task, which was similar to the tasks in previous studies [7, 12]. They listened to chord sequences and rated how well the last chord of each sequence belonged to the context perceptually, by pressing a button on a 9-point scale (1 = least appropriate; 5 = neutral; 9 = most appropriate). In this study, the rating value was referred to as a “Degree of Relatedness,” abbreviated as a DOR. The chords in each sequence were chosen from the 12 major triads. The sequences consisted of 2, 3, or 4 chords (For details, see Materials and Procedures). We analyzed the listeners’ DORs (see S1 Data) utilizing statistical and multivariate methods. We then built computational models based on this analysis, and compared their performance to approximate the obtained DOR data. An advantage of the present approach is that it does not assume internal representations of keys and tonal functions of chords (e.g., a dominant). This enables us to analyze the DORs of very short chord sequences to which Western music theory cannot attribute unique tonal functions. We nevertheless came to a conclusion that the simulated mechanism of harmonic expectancy included some important characteristics of Western music theory. As previous studies indicated, tonality played a critical role [14, 15, 26].

## Results

### Pre-modeling Analyses of Behavioral data

**Fig 1:**
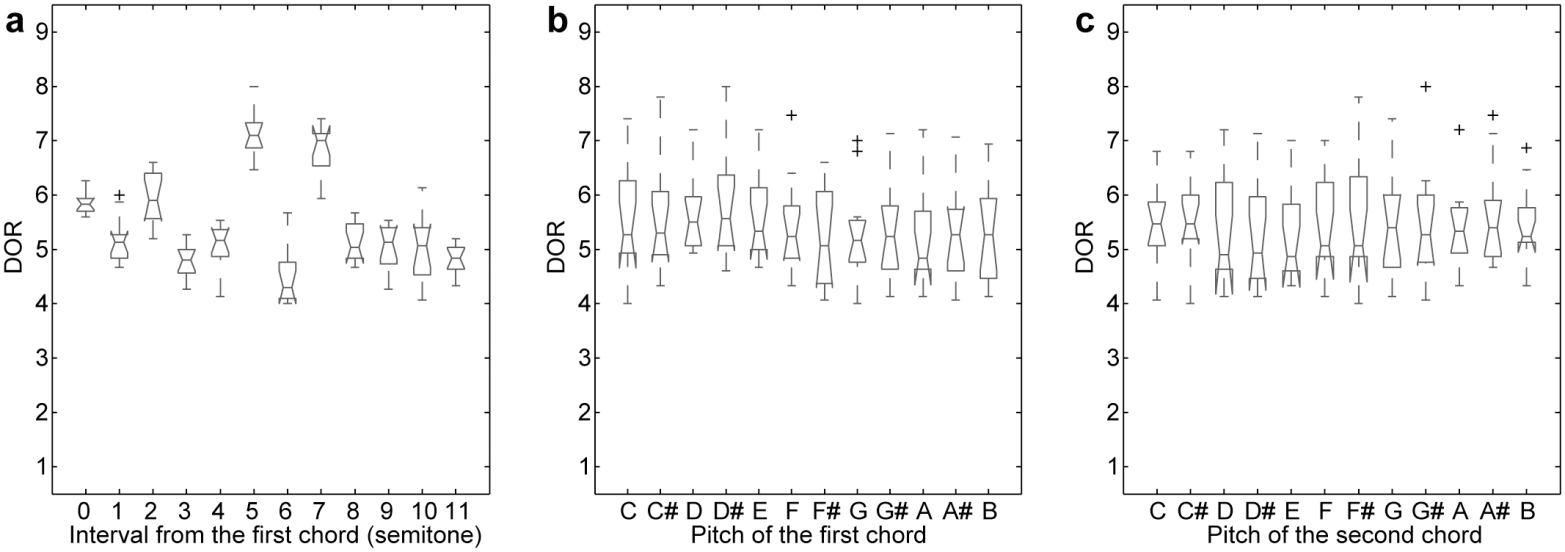
Averaged DORs in the 2-chord condition. Box-plots of the averaged DORs with respect to (a) the interval, (b) the first chord, and (c) the second chord.

**Fig 2:**
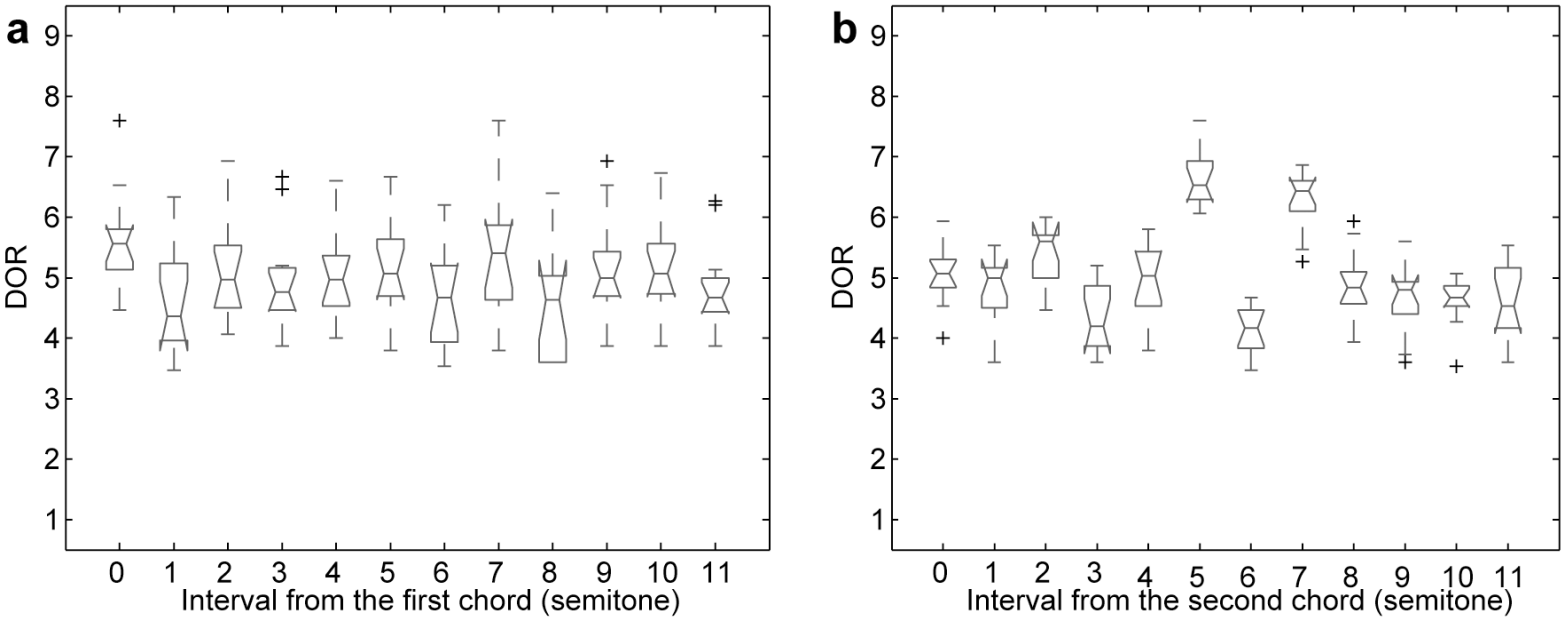
Averaged DORs in the 3-chord condition. Box-plots of averaged DORs with respect to the interval (a) from the first chord to the third chord, (b) from the second chord to the third chord.

Statistical analyses were performed for the averaged DORs across all participants for each chord context (see Figs 1, 2 and S1 Fig). For the 2-chord condition, three factors were tested: the pitch (tone chroma) of the root of the first chord, the pitch of the root of the second chord, and the interval between them. Results showed that the effect of the interval was significant (Fig 1; Kruskal-Wallis test, *χ*^2^(11) = 100.8; *p* < 0.001). The effects of the first-chord pitch (*χ*^2^(11) = 8.0; *p* = 0.71) and of the second-chord pitch (*χ*^2^(11) = 4.7; *p* = 0.95) were not significant. This shows that the interval is a critical factor for the DORs, but that the pitches are not.

In the 3-chord condition, two intervals were related to the target chord (i.e., from the first chord to the third chord and from the second chord to the third chord). Fig 2 presents box-plots of the DORs with respect to each interval. Friedman’s tests showed that there was a significant effect of each interval (interval from the first chord: *χ*^2^(11) = 41.7; *p* < 0.001, interval from the second chord: *χ*^2^(11) = 85.1; *p* < 0.001).

**Fig 3:**
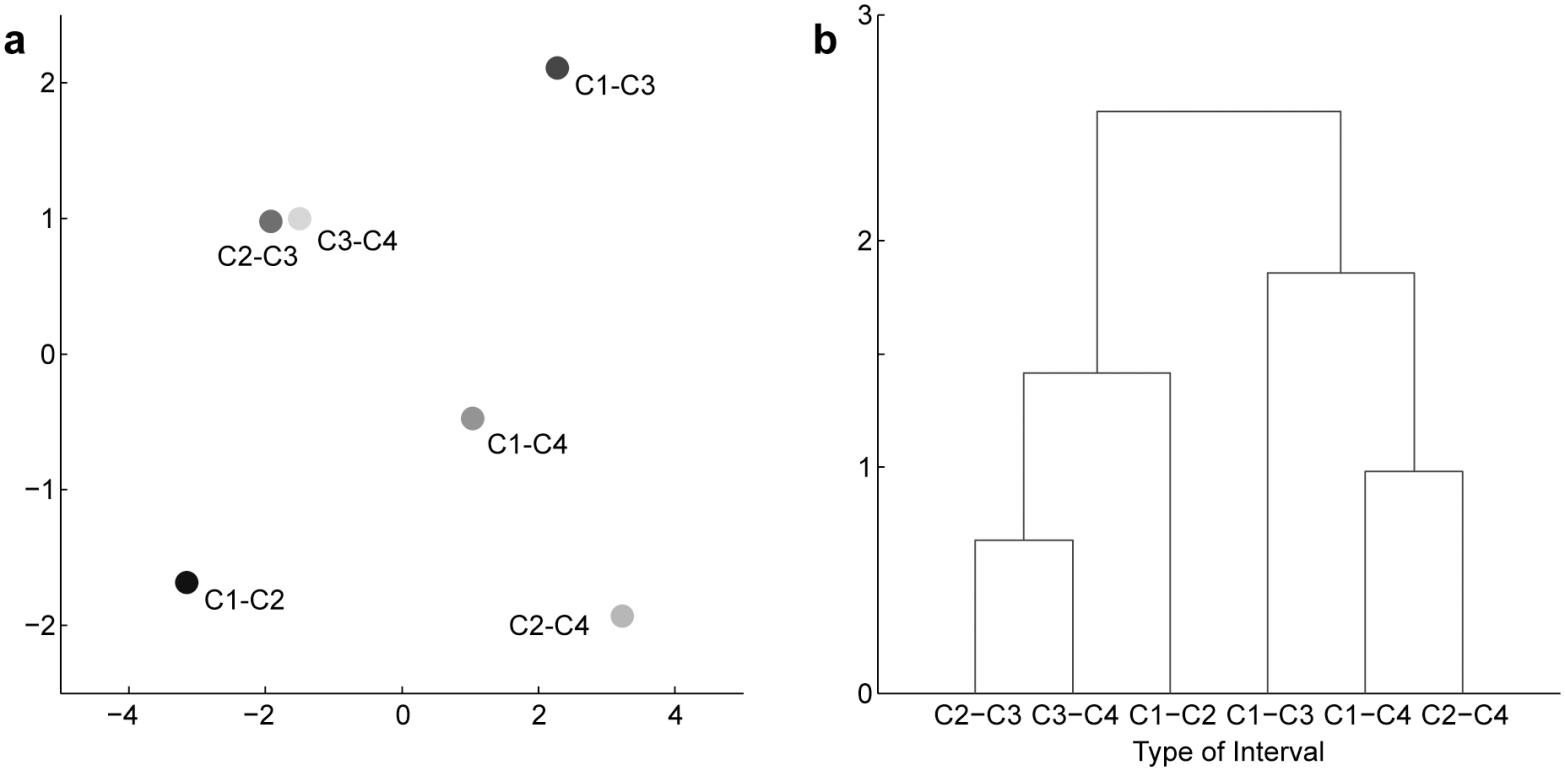
Grouping analyses of the DORs. (a) Result of non-metric multidimensional scaling analysis (in the two-dimensional case, *stress* < 0.001). Each circle corresponds to the vector of the averaged DORs. DORs averaged with respect to the interval between the roots of the *m*th chord and the *n*th chord (i.e., the last chord) are represented by C*m*-C*n*. Distance between the circles illustrates dissimilarity between chord-pair intervals. (b) A tree plot of the hierarchical clustering. Length of the connection path represents the distance of the DORs between corresponding intervals.

To examine the influence of the chord-to-chord intervals on the DORs, similarity between interval influences was examined by using grouping analyses. Our experiment included six chord positions preceding the target chord: one in the 2-chord condition, two in the 3-chord condition, and three in the 4-chord condition. We took up each of these chord positions. The interval between the chord in each position and the target chord was the focus of the following analysis. First, mean DORs were calculated for those intervals which were commonly included in the 2-, 3-, and 4-chord conditions (i.e., major second, fourth, fifth, and minor seventh degrees). Following this, a four-dimensional vector was constructed from the four mean DORs. Non-metric multidimensional scaling [27] was applied to the Euclidean distance matrix between each pair of the vectors (Fig 3a). The distance between the vectors corresponding to the interval from the second chord to the third chord in the 3-chord condition and the interval from the third chord to the fourth chord in the 4-chord condition was shorter than the distances between other paired vectors.

We also applied hierarchical clustering [28] to the same vectors of the mean DORs. The tree structure obtained by the complete-link method could be divided into two large groups (Fig 3b). The DORs revealed similar patterns when intervals between the last adjacent chords were the same (leading to the left cluster). These results suggest that the influences of the penultimate chords on the DORs were similar, particularly in the 3-chord condition and the 4-chord condition.

To investigate the influence of the chords preceding the penultimate chords, the DORs in the corresponding 2-chord conditions were subtracted from the DORs in the 3-chord condition (S2 Fig). When the third chord was identical to or seven semitones (i.e., fifth degree) above the first chord, the DORs showed significantly higher values than when the third chord was one or eight semitones (i.e., minor second or minor sixth degree) above the first chord (Friedman’s test, *χ*^2^(11) = 85.1; *p* < 0.001; mean-rank post-hoc test with Bonferroni correction, *N* = 12, *p* < 0.00076). This supports the idea that the first chord also plays an important role in harmonic expectancy for the last chord (i.e., the third chord).

### Computational-model-based analysis

Based on the results of the pre-modeling analyses, we modeled the computational process behind the subjective relatedness. The results of the behavioral judgments clarified a couple of issues about DORs. First, the *intervals* between the pitch of the last chord and the pitches of any preceding chords in a chord sequence determined the DOR, but the pitches themselves did not. Second, the strong influence of the interval between the last two adjacent chords was kept regardless of the chord-sequence length. These tendencies can be approximately expressed by introducing a Markov-chain-like dependence of the DORs on the pitch of the penultimate chord and a reference variable of intervals.

However, the influence of the other preceding chords was not consistent with a simple Markov chain assumption. There are two computationally feasible solutions to this problem. One is extension of the Markov chain (e.g., a variable-order Markov model), and the other is extension of the reference variable by assuming its own dynamics (e.g., a hidden Markov model). The latter extension lets the reference candidates have their probabilistic distribution, as a hidden variable which rules the pattern of DORs, and any pitch can be the internal reference.

We built several candidate models and compared their generalization performances. A *second-order Markov* model (2M model) corresponds to the chain extension model, and a *Bayesian updating* model (BU model) and a *Bayesian updating and switching* model (BS model) corresponds to the models with a hidden reference variable. In the BU model, a stochastic variable, i.e., a reference variable, is updated sequentially according to observation. The BS model is an extended model of the BU model, in which it can reset the reference variable based on its uncertainty. Performances of a few other models that could be related to the data were also examined. They were a *first-order Markov* model (1M model), a *pitch-based Markov* model (2P model), and a *pitch-based* model (1P model).

**Fig 4:**
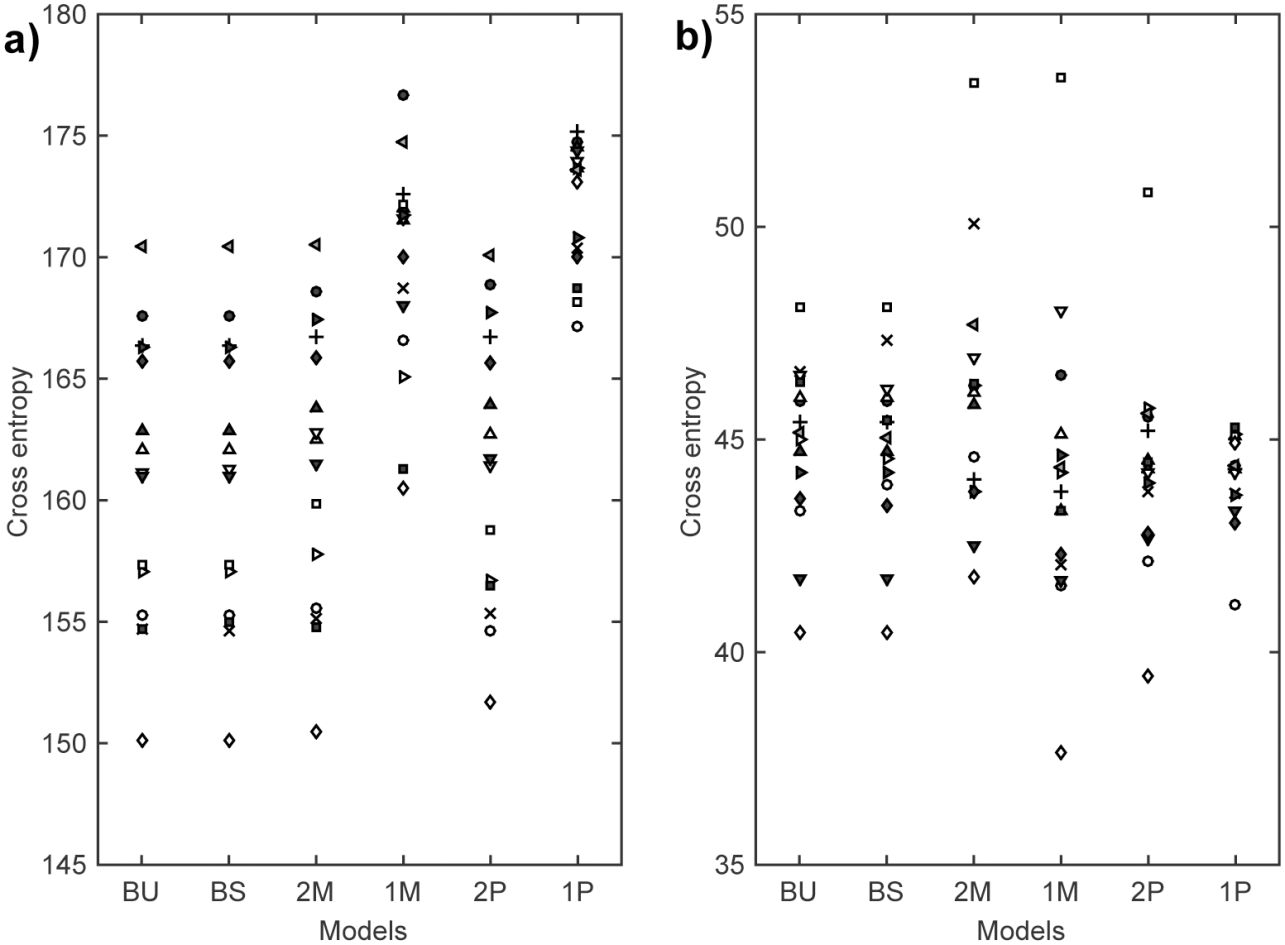
Performance comparison between models (individual data analysis). The cross entropies, which were obtained by Eq (13), for (a) the training data (i.e., in the 2-chord and in 3-chord condition) and (b) the test data (i.e., in the 4-chord condition) are plotted. The cross entropies of the same participant are represented by the same marker. A lower value indicates that the model can explain the data better.

The parameters were estimated by minimizing the cross entropy between the DORs and model outputs (i.e., a sum of conditional negative log-likelihoods given the DORs). Here, model estimation and evaluation were performed in two ways, i.e., by means of a comparison of individual data and by a group analysis with a cross-validation method. For the individual data analysis, each model was trained by the DORs in the 2-chord and 3-chord conditions. Following this, the generalization performances of the trained models were confirmed by the DORs in the 4-chord condition. Fig 4a and 4b show the cross entropies for the training data and the test data, respectively. A summary of statistical tests of the cross entropies for the test data is shown in Table 1. Both the BU and the BS model revealed significantly lower values than the 2M model (sign test, *p* < 0.05). The reference extended models approximated the participants’ responses better than the 2M model. There were no significant differences between the cross entropies of the BU and the BS model. The simple Markov model (1M model) and the pitch-based models (1P and 2P model) also showed low values for the test data.

**Table 1:** Summary of statistical tests in the individual analysis.

		sign test			
model 1	model 2	positive difference 1-2	negative difference 1-2	*N*	*p*-value
BU	BS	5	2	7	0.453
BU	2M	3	12	15	0.035*
BU	1M	11	4	15	0.119
BU	2P	11	4	15	0.119
BU	1P	11	4	15	0.119
BS	2M	2	13	15	0.007**
BS	1M	11	4	15	0.119
BS	2P	11	4	15	0.119
BS	1P	11	4	15	0.119
2M	1M	11	4	15	0.119
2M	2P	12	3	15	0.035*
2M	1P	12	3	15	0.035*
1M	2P	5	10	15	0.302
1M	1P	6	9	15	0.607
2P	1P	9	6	15	0.607

(* for *p* < 0.05,

** for *p* < 0.01)

**Fig 5:**
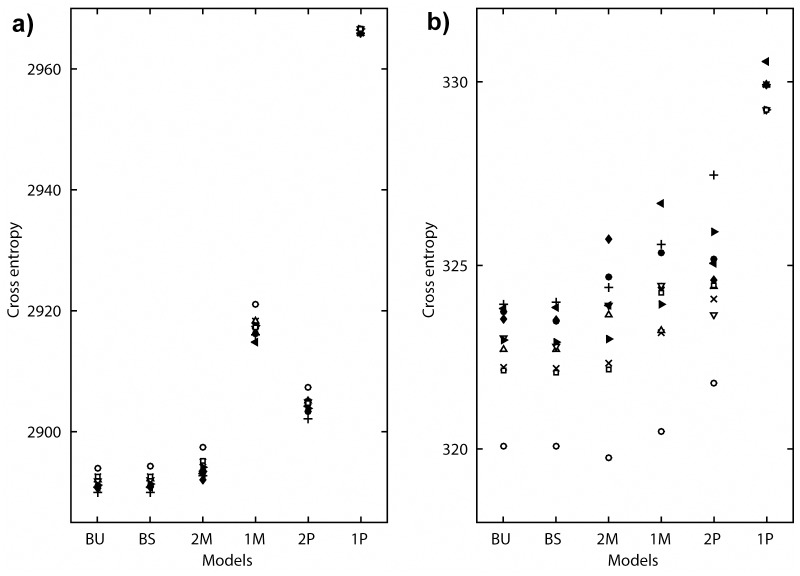
Performance comparison between models (10-fold cross-validation). Cross entropies for (a) the training subsets and (b) the test subsets are plotted. The values from the same samples are represented by the same marker.

**Fig 6:**
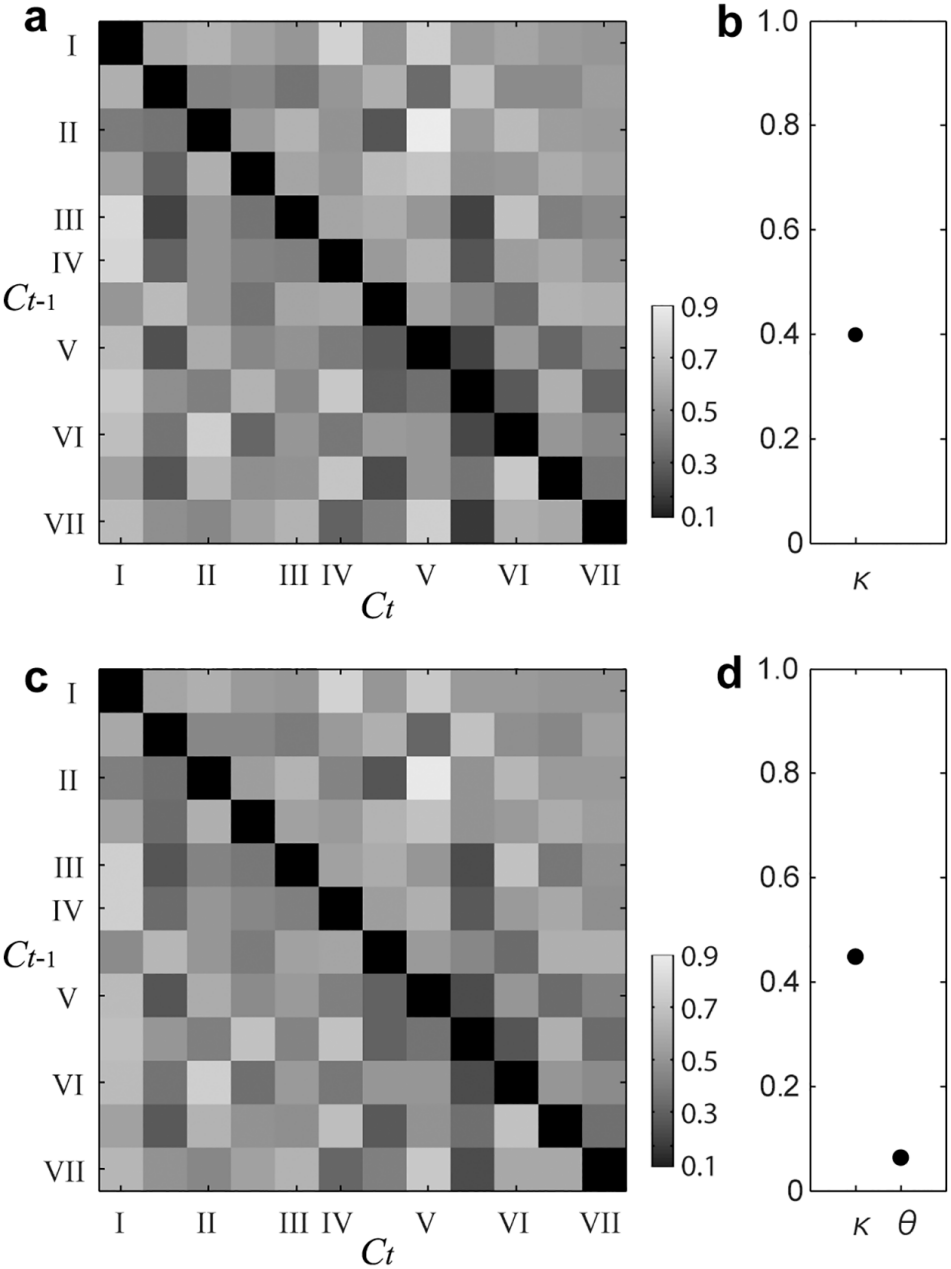
Estimated parameters. Upper panels (a-b) are the results of the BU model, and lower panels (c-d) are the results of the BS model, obtained by the cross validation analysis. (a) and (c) are estimated expectancy matrices (i.e., $\hat{\text{T}}$), which show subjective relatedness of corresponding chord progression when a reference pitch, I, is given. Axes are labeled by the intervals from the reference. Gray scale represents the estimated value. Here, self-transitions are excluded (orthogonal blacked squares). (b) and (d) show the other estimated parameters (i.e., $\hat{\kappa }$ and $\hat{\theta }$), which are an initial weight of the first chord for the pitch center and a threshold for resetting the prior probability of the reference, respectively). In the BU model, *θ* is always 0 (no value). See also S3 and S4 Figs, which are the individual estimated parameters of the BU and the BS model, respectively. See Computational Models, for details of each parameter.

Since complexities were different among the models, a cross-validation method with data from all conditions was necessary to evaluate the generalization performances. In order to avoid bias of the limited number of samples from each participant, we applied cross-validation to the data from all conditions and all participants. Results of the group analysis with a 10-fold cross-validation are shown in Fig 5 and Table 2. In agreement with the results of the individual data analysis, the BU and the BS model showed significantly lower cross entropies than the 2M model, and also than the 2P model and the other models (*p* < 0.05 with sign tests for all combinations). There was no significant difference between the BU and the BS model. The means of estimated parameters of the BU model are shown in Fig 6a and 6b, and those of the BS model are shown in Fig 6c and 6d.

**Table 2:** Summary of statistical tests in the group analysis.

		sign test				paired T-test		
model 1	model 2	positive difference 1-2	negative difference 1-2	*N*	*p*-value	*T*	*N*	*p*-value
BU	BS	6	4	10	0.754	1.570	10	*p* = .151
BU	2M	1	9	10	0.022*	−2.343	10	*p* = .044*
BU	1M	0	10	10	0.002**	−5.595	10	*p* < .001***
BU	2P	0	10	10	0.002**	−6.628	10	*p* < .001***
BU	1P	0	10	10	0.002**	−22.433	10	*p* < .001***
BS	2M	1	9	10	0.022*	−2.447	10	*p* = .037*
BS	1M	0	10	10	0.002**	−5.702	10	*p* < .001***
BS	2P	0	10	10	0.002**	−7.215	10	*p* < .001***
BS	1P	0	10	10	0.002**	−23.511	10	*p* < .001***
2M	1M	2	8	10	0.109	−2.201	10	*p* = .055
2M	2P	2	8	10	0.109	−3.029	10	*p* = .014*
2M	1P	0	10	10	0.002**	−13.661	10	*p* < .001***
1M	2P	3	7	10	0.344	−1.366	10	*p* = .205
1M	1P	0	10	10	0.002**	−12.037	10	*p* < .001***
2P	1P	0	10	10	0.002**	−11.680	10	*p* < .001***

(* for *p* < 0.05,

** for *p* < 0.01,

*** for *p* < 0.001)

## Discussion

To investigate the mechanism underlying harmonic expectancy, we conducted a behavioral experiment in which participants rated the subjective Degree of Relatedness (DOR) of chord progressions. The data were analyzed with a computational-model-based approach. The results showed that the DORs are determined by the interval between the penultimate and the final chord as well as an internal reference to be updated. The reference takes multiple states, corresponding to pitches, and has its own distribution. The balance of states is updated sequentially, based on the likelihood of the observation.

This process may correspond to what Western music theory describes: When a chord sequence is listened to, tonality is constructed dynamically, and it dominates the harmonic expectancy for the subsequent chord. This is in line with previous studies indicating the consistency between the harmonic expectancy and the prediction from the tonal functions of the chords [8, 12, 18]. In Western music theory, the tonal function is ruled by the key of the presented sequence. An additional assumption is the Markov modeling of root transition in a certain key [29]. In this respect, our proposed models of harmonic expectancy are consistent with Western music theory. Analyses based on Western music theory, however, cannot be applied to the chord sequences as in our paradigm, since most of the present sequences were too short to lead to unique keys. Nevertheless, the stochastic analyses that were central to the computational processes provide evidence that a kind of tonality concerns the subjective relatedness of the chord progression.

The contribution of the reference in our models is also consistent with the spread activation model [17]. Weights of linkages between the chord layer and key layer in the spread activation model correspond to the expectancy matrix and implicitly act to emphasize connections between related chords, like a Markov transition of chords in our suggested models (i.e., BU and BS models).

Both BU and BS models, which performed best for the present data, include Bayesian updating of knowledge about the reference. The Bayesian framework can derive an optimal posterior distribution of the reference when a likelihood of the observation (i.e., an expectancy matrix) and prior knowledge (i.e., prior distribution of the reference) are given. Bayesian integration was reported in functional models of various types of brain functions and perceptual abilities, like sensorimotor control [30], vision [31], temporal order judgment [32], time—interval perception [33], and decision making [34, 35]. A Bayesian model that assumed an informational hierarchy of musical scores performed well in automatic key-finding problems [25]. These previous studies support the idea that the present participants guessed the reference (i.e., the key) from a stochastic musical structure of chord sequences according to Bayesian-like processes.

Expectancy matrices in the BU and the BS model (see Eq (5)) show how much a chord progression fits to the reference. The estimated matrices obtained by the DORs from all participants agree with expectations from Western music theory (see Fig 6a and 6c); the fitness of popular progressions (e.g., II to V) show high scores, while the fitness of rarer progressions (e.g., II to IV) showed lower scores (see S1 Table, for more examples). Note that all chords utilized in the present study are major triads. Although there were individual differences in the estimated expectancy matrices (see the individual results in S3 and S4 Figs), the criterion of the rating scale was also different between participants.

**Fig 7:**
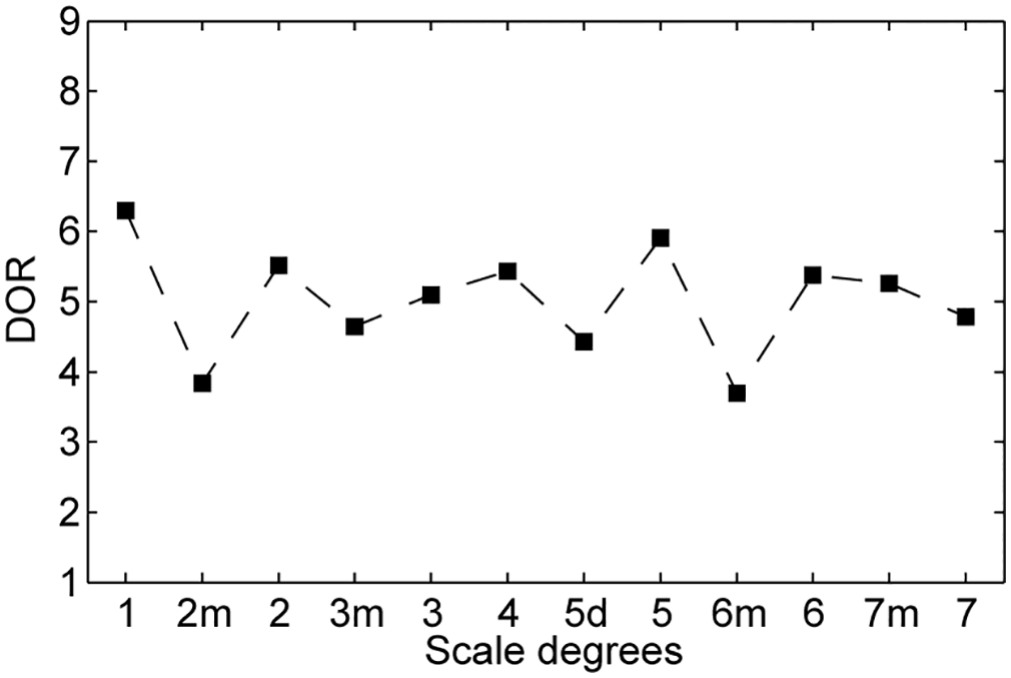
Key-profile obtained by the expectancy matrix of BU model. The horizontal axis shows the root of major triads which is labeled by the interval from the reference. The vertical axis represents the ideal DOR when the unique reference is given.

To relate the present results with previous research, we computed a key-profile from the estimated matrix (Fig 7). The obtained profile was similar to the profiles obtained by the probe-tone method [1, 36] as well as to the profiles derived from score analyses [25]. The pattern of the harmonic expectancy is often considered to be learned unconsciously [11, 17], and is similar to the frequencies of the chord progression in Western music [37, 38]. Actually, some non-typical progressions (e.g., V♯ to IV, VI♯ to VI) showed higher probability than V to I, which is also a popular progression in tonal music [29]. Since the chords in the non-typical progressions include non-diatonic note(s), Western music theory does not directly cover such progressions. These findings suggest that Western music theory can be extended with more general chord progressions from a viewpoint of perceptual psychology.

The listener’s prior knowledge when the first chord is given, is represented by a weight parameter *κ* (Eq (6)) in the BU and the BS model. The estimated values of *κ* were always much higher than 1/12 (Fig 6b and 6d), indicating that the participants tended to perceive the first chord as a reference (i.e., a tonic) of the coming context.

With regard to the reference, we established a switching parameter *θ* in the BS model (Eq (7)). *θ* enables to reset the knowledge of the reference (i.e., key information) when tonal uncertainty was too high, like in a “key change [39]”. Since the BS model is an expanded version of the BU model, its performance should be the same or better than that of the BU model. When *θ* is equal to 0, the BS model is identical to the BU model. The present study shows that the model performance of the BS model was not much better than that of the BU model in both the individual analysis (Fig 4b) and the cross-validation (Fig 5b). In this study, the maximum length of the chord sequence was only four chords. This might have been a disadvantage for the BS model, since a switching event (i.e., a key change) was unlikely to occur.

In summary, the fitness of chord progression seemed to be influenced by the updated tonal reference. In principle, our method can be applied not only to major triad sequences, but to any types of stimuli. It is necessary, in future work, to test whether the BU or the BS model works for longer chord sequences, for other types of chords, and for any other duration of chords. Modeling with a small amount of behavioral data is difficult, and the computational-model-based approach presented here turned out to be an effective way to examine interactions that include internal (i.e., hidden) variable(s). Western music theory, with score analyses, provides important cues [38, 40]. The computational-model-based approach will give us new insights into the mechanism of music perception.

## Methods

### Ethics statement

The procedures were reviewed and approved by the ethical committee of the Faculty of Design, Kyushu University. All participants were explained the task design and gave written informed consent before starting the experiment.

### Participants

Fifteen healthy adults (nine males and six females, age 24.9±4.0) had participated in the experiment. Participants had normal hearing and had 10.1±5.7 years of musical training in addition to Japanese compulsory education including music.

### Materials and Procedures

The chords in each sequence were selected from the twelve major triads, and had durations of 0.5 s. The sequences consisted of 2, 3, or 4 chords. For the 2-chord condition, all permutations of the chords were presented. The number of trials was 144. For the other conditions, since the number of permutations would be very large, we just took up intervals between the roots of the sequential triads, while initial chords were chosen at random. For the 4-chord condition, only sequences consisting of four types of intervals (major second, perfect fourth, perfect fifth, and minor seventh degree) were used. Thus, the numbers of trials for the 3-chord condition and the 4-chord condition were 144 and 64, respectively.

The presented chords consisted of pure tones in twelve-tone equal temperament with A4 = 440 Hz as the reference tone. To avoid too obvious effects of inversion and voicing, the tones in each chord were sounded over a three-octave range (from G3 to F♯6) and the intensity of higher and lower tones from its middle one-octave range were decreased with the pitch distance [41], in a similar way to that of Krumhansl, Bharucha, and Kessler (1982) [7].

The participant was seated in a chair facing to a computer display. The chord sequences were presented diotically from headphones (STAX SR-307) with an amplifier (STAX SRM-323S), and the level of the sounds was adjusted to 60 dB (A) as measured with an artificial ear (Brüel and Kjær 4153), a microphone (Brüel and Kjær 4134), and a sound level meter (Nagano Keisoku 2071).

For each trial, a white cross cue appeared on the middle of the display 1000 ms before the sound presentation. The participant was instructed to judge the degree of relatedness (DOR) of the last chord, in the context of the current chord sequence, by pressing a button as soon as possible after the onset of the last chord. The cue disappeared immediately after the judgment. A rest period of 1500 to 3000 ms preceded the next trial. The stimulus presentation and recording of button responses were conducted by using Cogent toolbox (Wellcome Department of Imaging Neuroscience: http://www.vislab.ucl.ac.uk/Cogent) for Matlab (Mathworks).

The experiment was divided into three sessions, which were performed in the order of the 2-chord, the 3-chord, and the 4-chord condition. Before each session, the participant was told the type of condition. The participant performed 20 warm-up trials. The warm-up stimuli were chosen at random. The total duration of the experiment, including the instructions and the rests, was about one and half hours.

### Computational Models

#### Stochastic Modeling of Harmonic Expectancy

We modeled a computational process underling the relatedness judgments by assuming the results of the pre-modeling analyses.

Now, consider that one chord is observed at each time step *t*. While *t* ≥ 2, the internal fitness of the target chord, c, can be described by using conditional probability,

$$\begin{array}{c} E\left(c_{t}=c\right)=P\left(c_{t}=c|c_{1:t-1}\right), \end{array}$$ (1)

where *c*_*t*_ is the chord observed at *t*, and *c*_1: *t*−1_ represents all past observations in the current chord sequence. In our experiment, candidates of the chord were twelve types of major triads.

$$\begin{array}{c} c_{t}\in \left\{A\;\text{major},A♯\;\text{major},B\;\text{major},C\;\text{major},\ldots ,G♯\;\text{major}\right\}. \end{array}$$ (2)

To express the dependence of the fitness on intervals between the pitches of the chords, let a stochastic variable *X* be the internal reference of the interval. Here, *X* can take all twelve triads:

$$\begin{array}{c} X\in \{A\;\text{major},A♯\;\text{major},B\;\text{major},C\;\text{major},\ldots ,G♯\;\text{major}\}. \end{array}$$

Eq (1) is modified by introducing *X* as follows:

$$\begin{array}{c} E\left(c_{t}=c\right)=\sum_{X}P\left(c_{t}=c|X,c_{1:t-1}\right)P\left(X\right). \end{array}$$ (3)

The strong influence of the interval from the penultimate chord can be described by introducing a Markov chain-like dependence in the likelihood:

$$\begin{array}{c} E\left(c_{t}=c\right)=\sum_{X}P\left(c_{t}=c|X,c_{t-1}\right)P\left(X\right). \end{array}$$ (4)

However, a simple Markov assumption generally cannot explain the dependence of the listener’s judgment on the other preceding intervals.

#### Bayesian updating model (BU)

One possible solution of describing the influence of preceding non-adjacent intervals is to assume a dynamic update of the distribution of *X* depending on the observation. An optimal updating equation of the conditional probability of *X* given the context can be derived by using Bayes’ rule as follows:

$$\begin{array}{c} P\left(X|c_{1:t}\right)\propto P\left(c_{t}|X,c_{t-1}\right)P\left(X|c_{1:t-1}\right). \end{array}$$ (5)

Here, the likelihood, which corresponds to a transition matrix of Markov model, is parameterized by an expectancy matrix, **T**. Each element in the matrix, **T**(*i*, *j*), corresponds to a transition probability (i.e., harmonic expectancy) of going from a preceding chord *i* to a current chord *j* when *X* is given. Since there are 12 types of triads in this study, **T** includes 144 types of chord progressions by combining two triads. It is notable that each chord is not labeled by its pitch, but by an interval from the reference, *X*. In other words, *X* can perform like a tonal center in Western music theory. For example, if *X* is matched to A♯, a chord of D♯ will be labeled as 6, five semitones above *X*, so that the transition probability of going from D♯ to A♯ will be shown in **T**(6, 1).

An initial state of *X* is modeled by introducing a parameter, *κ*.

$$\begin{array}{c} P\left(X|c_{1}\right)=\left\{\begin{array}{cc} \kappa & (X=c_{1}),\\ \frac{1-\kappa }{11} & (\text{otherwise}), \end{array}\right. \end{array}$$ (6)

where 0 < *κ* ≤ 1. From this parameterization, it is possible to infer the influence of the first observation on the establishment of the reference.

By updating *X* based on Eq (5), an optimal posterior distribution can be obtained. We referred to this ideal model as the Bayesian updating model (BU model). The BU model holds twelve candidates of the reference and sequentially updates their balance by evaluating the observation.

#### Bayesian updating and switching model (BS)

The BU model can be extended by focusing on the property of the conditional probability of *X*. When an observed chord is unexpected, the conditional probability of *X* will be lower. By introducing a threshold of the probability, *θ*, it is possible to update the reference quickly and adaptively according to its uncertainty. Here, the Bayesian updating and switching model (BS model) will reset and re-calculate the distribution of *X* when the conditional likelihood is lower than a given threshold:

$$\begin{array}{c} P\left(X|c_{1:t}\right)\propto \left\{\begin{array}{cc} P\left(c_{t}|X,c_{t-1}\right)P\left(X|c_{t-1}\right) & (t=2\;\text{or}\;P\left(X|c_{1:t}\right)<\theta ),\\ P\left(c_{t}|X,c_{t-1}\right)P\left(X|c_{w:t-1}\right) & (\text{otherwise}), \end{array}\right. \end{array}$$ (7)

where *w* is the recent time step of switching the prior probability of *X*. For example, *w* = 1 when no switch occurred.

#### Second-order Markov model (2M)

Another possible extension of the Markov model is a multi-order Markov model. The results of multidimensional scaling and hierarchical clustering showed that the influences of the interval from the second chord to the third chord and the interval from the third chord to the fourth chord are similar (see Fig 3). This pattern can be reproduced by a second-order Markov model (2M model). For *t* ≥ 3, Eq (3) is modified as follows:

$$\begin{array}{c} E\left(c_{t}=c\right)=\sum_{X}P\left(c_{t}=c|X,c_{t-1},c_{t-2}\right)P\left(X\right). \end{array}$$ (8)

In this model, the reference is always fixed to *c*_*t*_ through the context. Thus the expectancy matrix **T** will be 144 combinations of two intervals; each element **T**(*i*, *j*) corresponds to a transition probability of going to *c*_*t*_ after observing *i* semitones lower than *c*_*t*−2_ and *j* semitones lower than *c*_*t*−1_. For *t* = 2, we assume a flat prior so that the expectancy matrix will be averaged with respect to *c*_*t*−2_.

#### Other models

Other models were also examined.

The simplest model is a pitch-based model (1P model), in which the DOR is determined by the pitch of the current triad:

$$\begin{array}{c} E\left(c_{t}=c\right)=P\left(c_{t}=c\right). \end{array}$$ (9)

By considering the influence of the penultimate chord, a pitch-based Markov model (2P model) can be defined. For *t* ≥ 2, Eq (3) is modified as follows:

$$\begin{array}{c} E\left(c_{t}=c\right)=P\left(c_{t}=c|c_{t-1}\right). \end{array}$$ (10)

The first-order Markov model (1M model) was also a candidate. Note that the 1M model is a simplified case of the 2P model, in which the diagonal elements of the expectancy matrix is reduced to take the same value.

#### Parameter Estimation

The parameters were optimized to explain the behavioral responses. When the participant listened to the last chord of the sequence, the response of DOR was:

$$\begin{array}{c} A\left(c_{\tau }\right)\in \left\{1,2,3,4,5,6,7,8,9\right\}, \end{array}$$

where *τ* is the index of the last chord. Here, a simple linear relationship is assumed between *A* and *E* as follows:

$$\begin{array}{c} A\left(c_{\tau }\right)=10\cdot E\left(c_{\tau }\right). \end{array}$$ (11)

This assumption seems sufficient to compare model performances and to discuss the tendencies of each estimated parameter; it satisfies two important characteristics of a probability, 0 ≤ *E*(*c*_*τ*_)≤1 and *E*(*c*_*τ*_) = 0.5 when the relatedness of the chord progression is neutral, namely *A*(*c*_*τ*_) = 5.

For each model (and also each participant in the individual analysis), now the cross entropy between the DORs of the participants and the model outputs is:

$$\begin{array}{c} \left(\hat{T},\hat{\kappa },\hat{\theta }\right)\approx \underset{T,\kappa ,\theta }{\text{arg}\;\text{min}}H_{\left(A^{\prime },E\right)}, \end{array}$$ (12)

$$\begin{array}{c} H_{\left(A^{\prime },E\right)}\equiv -\sum_{n}\sum_{c_{\tau }^{n}}A^{\prime }\left(c_{\tau }^{n}\right)\log E\left(c_{\tau }^{n}|T,\kappa ,\theta \right), \end{array}$$ (13)

where *A*′ = *A*/10 and *n* is an index of trial (*n* = 1, 2, 3, …, *N*).

We first calculated the cross entropy with initial parameters. We then picked up a parameter in random order and updated it in the positive or negative direction to minimize the cross entropy as a cost function. All parameters were updated until the cost would not change any more. To avoid a local minimum, this procedure was repeated 100 times by changing the initial values at random. The parameters corresponding to the minimum cost were recorded as results. The minimum updating widths for each element of **T**, *κ*, and *θ* were 0.1, 1/12, and 0.05, respectively. Since ratings for successive identical chords were unstable, estimations of corresponding elements (e.g., diagonal elements of the expectancy matrix) were omitted in the analysis.

#### Model Evaluation

The cross entropy obtained from the parameter estimation indicates how well the model can explain the data. However, it is difficult to compare the cross entropies directly between the models, because the complexities of the models were not unified. Some correction methods using the information criterion (e.g., [42, 43]) were not feasible in our study, since the behavioral responses were acquired not by two-alternative forced choices but by rating scales; we assumed a simple linear relationship between the 9-point rating scale and the probability (see Eq (11)), and this assumption might cause imbalance between the log-likelihood and the cost term in the criterion.

We thus divided the data into two subsets: the training data, which consisted of data from the 2-chord condition and the 3-chord condition, and the test data, which were from the 4-chord condition. We estimated the model parameters from the training data, and then calculated the cross entropy for the test data. Additionally, a 10-fold cross-validation was performed; the data were randomly divided into 10 subsets, then 9 subsets and the remaining subset were utilized as training data and test data, respectively. This process was repeated for all 10 combinations of the subsets. Since the data size from each participant was small, data from all participants and all conditions (including the 4-chord condition) were utilized for this analysis.

#### Simulation Test

A simulation was performed to confirm the precision of parameter estimates. At first, based on a random parameter set and observation sequences, simulated outputs, corresponding to the DORs, were obtained for each model. The sequences were 253 patterns from the 2-chord and the 3-chord condition, which did not include consecutive chords. After that, in the same way as in the behavioral analyses, the model parameters were re-estimated from the observation sequences and the simulated DORs. We repeated these processes 100 times and calculated the mean bias and the standard deviation for each parameter set. See S5 Fig.

#### Key-profile

For the BU model, we simulated a key-profile based on its estimated expectancy matrix. While the original key-profile [36] represents the fitness of each pitch-class relative to each key, the key-profile of the BU model represents the DOR of each pitch-class relative to each pitch of the reference. We computed the key-profile, *P*(*c*_*t*_|*X*), by marginalizing the variable of the preceding chord,

$$\begin{array}{c} P\left(c_{t}|X\right)=\sum_{c_{t-1}}P\left(c_{t}|X,c_{t-1}\right). \end{array}$$ (14)

## Supporting Information

S1 Data DORs obtained in the experiment. Chords are labeled by numbers; A major triad is 1, A sharp major triad is 2, B major triad is 3,…, G sharp major triad is 12.
(CSV)

S1 Fig Averaged DORs across participants. (a) DORs in the 2-chord condition. Axes are indexed by pitches of roots of triads. Values of each condition are indicated in gray scale. (b) DORs in the 3-chord condition. Axes are indexed by intervals between chords. (c-f) DORs in the 4-chord condition, divided by the third interval (i.e., between the third and fourth chord); major second, fourth, fifth, and minor seventh degree are displayed in C, D, E, and F, respectively. Axes are indexed by intervals. M2th: major second degree, 4th: fourth degree, 5th: fifth degree, m7th: minor seventh degree.
(TIF)

S2 Fig Difference between the DORs of the 2-chord and 3-chord condition. The DORs of the 2-chord condition were subtracted from the corresponding DORs of the 3-chord condition. Positive and negative values mean increase and decrease of the DORs in the 3-chord condition, respectively.
(TIF)

S3 Fig Individual estimated parameters of the BU model. The estimated expectancy matrices in gray scale and box-plots of *κ* are shown.
(TIF)

S4 Fig Individual estimated parameters of the BS model. The estimated expectancy matrices in gray scale and box-plots of *κ* and *θ* are shown.
(TIF)

S5 Fig Simulated error of the parameter estimation. Each bar shows the mean error of the corresponding parameter. Error bars indicate standard deviations. A cross-mark indicates that there is no corresponding parameter. Note that, for the models which did not include the updating process of the reference, the error should not occur logically.
(TIF)

S1 Table Probability of the top 10 and bottom 10 progressions in the expectancy matrix of the BU model. Typical progressions in tonal music are marked by asterisks.
(CSV)
