# Supplementary figures and images for: Computational-Model-Based Analysis of Context Effects on Harmonic Expectancy
Source: PLoS One. 2016 Mar 22;11(3):e0151374. doi: 10.1371/journal.pone.0151374 (PMC4803284; doi:10.1371/journal.pone.0151374)

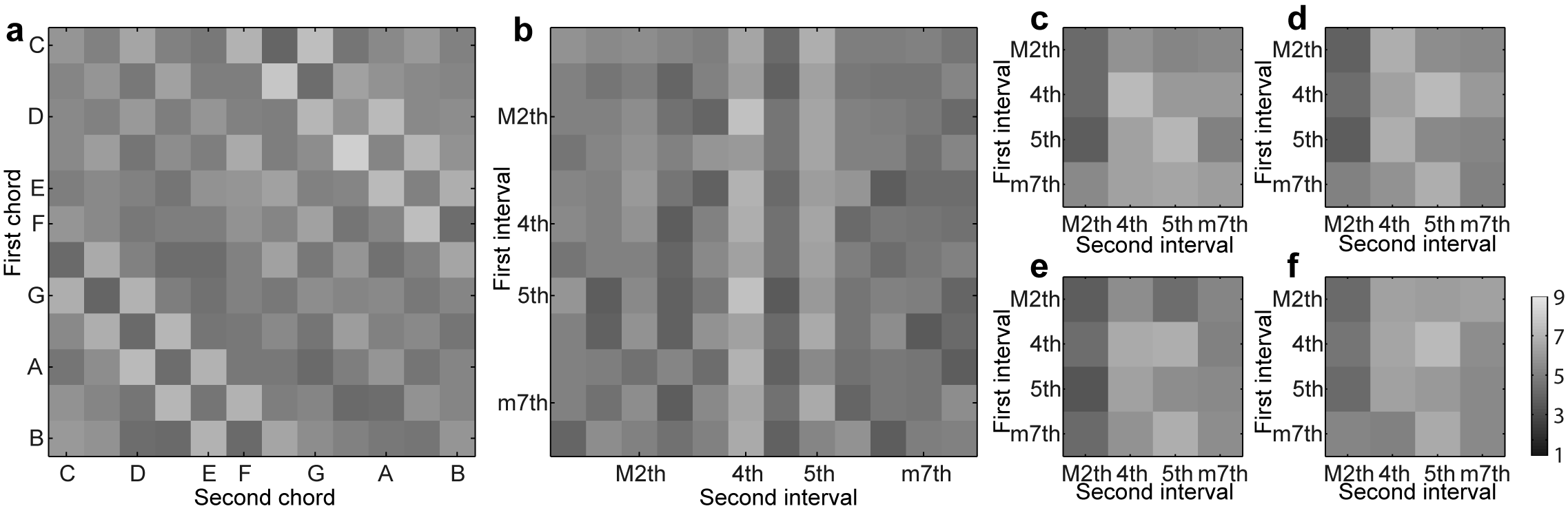

Supplement: S1 Fig — (a) DORs in the 2-chord condition. Axes are indexed by pitches of roots of triads. Values of each condition are indicated in gray scale. (b) DORs in the 3-chord condition. Axes are indexed by intervals between chords. (c-f) DORs in the 4-chord condition, divided by the third interval (i.e., between the third and fourth chord); major second, fourth, fifth, and minor seventh degree are displayed in C, D, E, and F, respectively. Axes are indexed by intervals. M2th: major second degree, 4th: fourth degree, 5th: fifth degree, m7th: minor seventh degree. (TIF) [file pone.0151374.s002.tif]

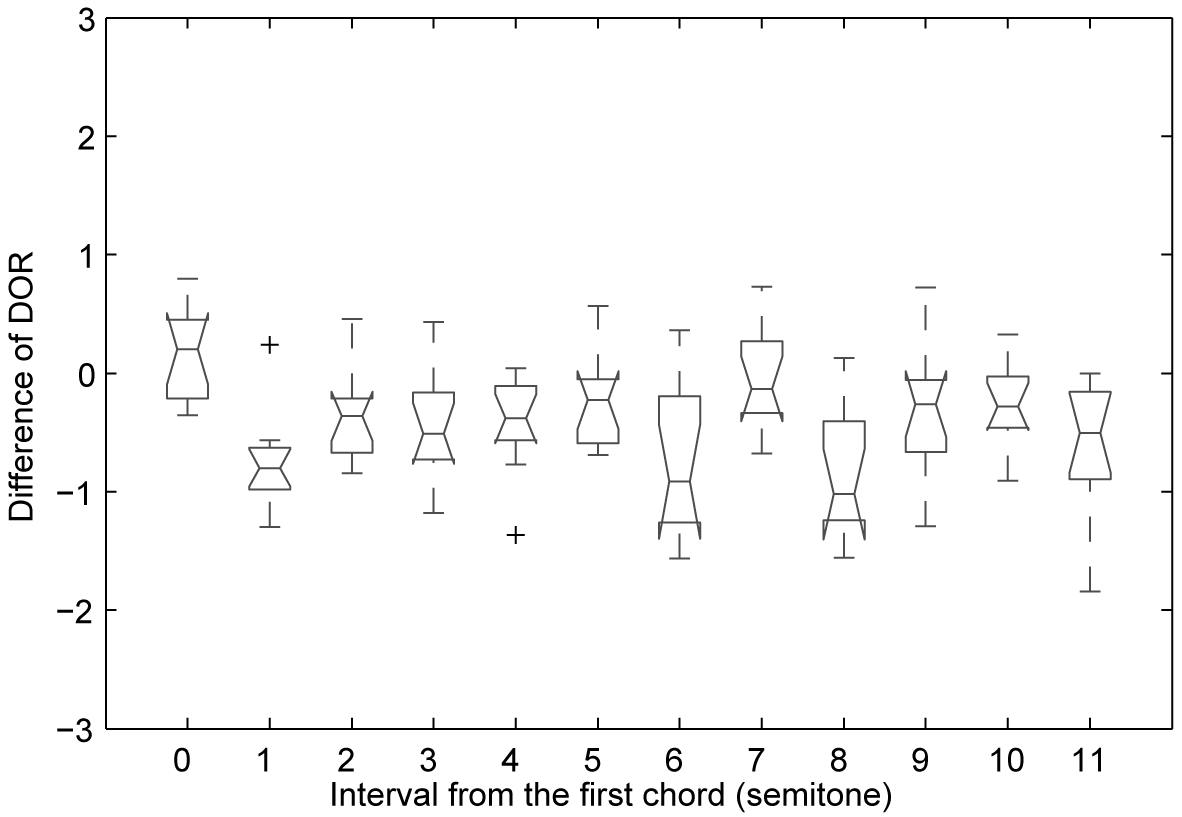

Supplement: S2 Fig — The DORs of the 2-chord condition were subtracted from the corresponding DORs of the 3-chord condition. Positive and negative values mean increase and decrease of the DORs in the 3-chord condition, respectively. (TIF) [file pone.0151374.s003.tif]

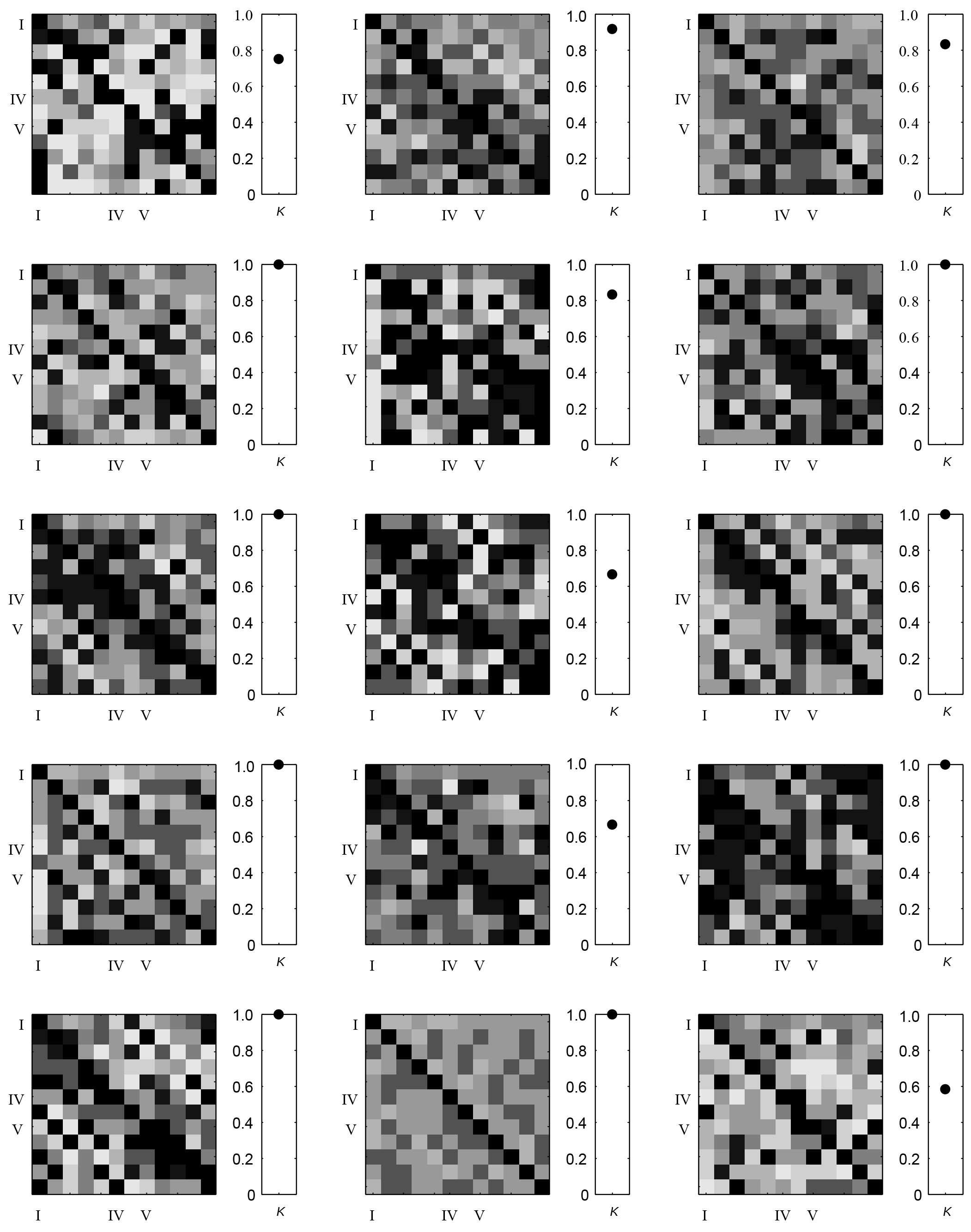

Supplement: S3 Fig — The estimated expectancy matrices in gray scale and box-plots of κ are shown. (TIF) [file pone.0151374.s004.tif]

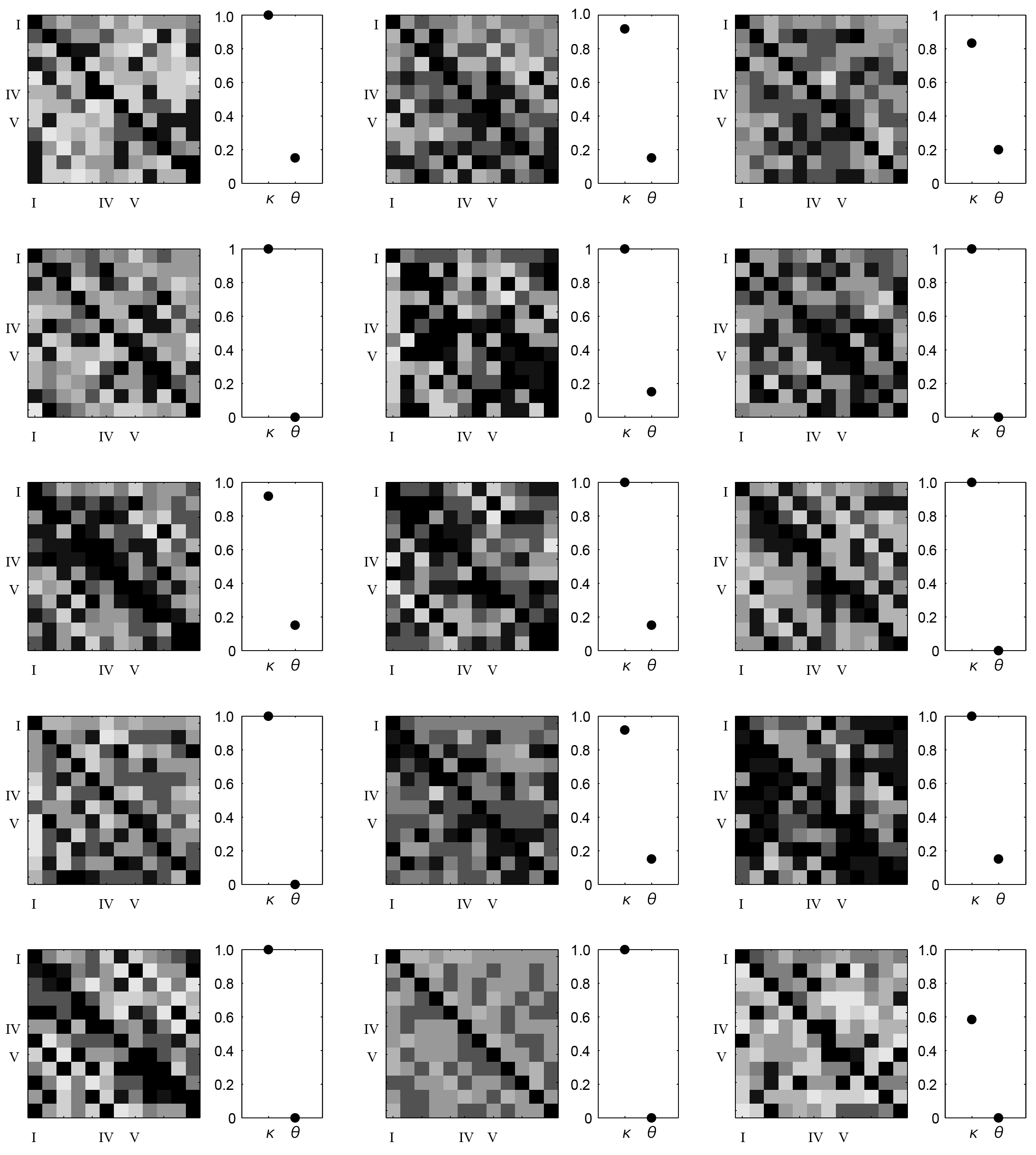

Supplement: S4 Fig — The estimated expectancy matrices in gray scale and box-plots of κ and θ are shown. (TIF) [file pone.0151374.s005.tif]

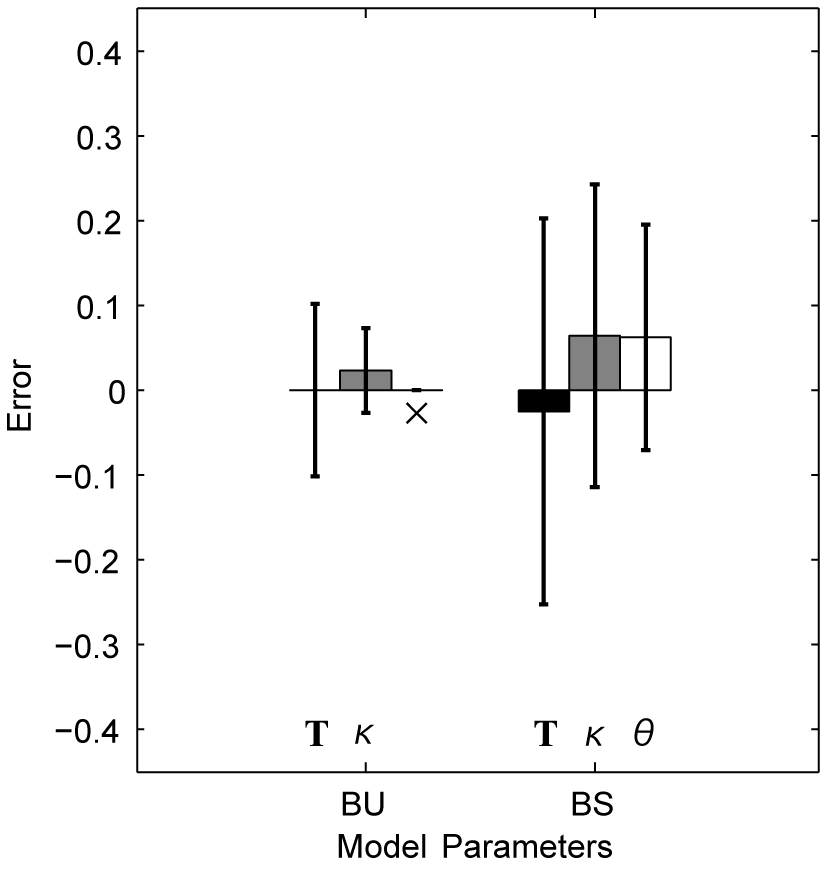

Supplement: S5 Fig — Each bar shows the mean error of the corresponding parameter. Error bars indicate standard deviations. A cross-mark indicates that there is no corresponding parameter. Note that, for the models which did not include the updating process of the reference, the error should not occur logically. (TIF) [file pone.0151374.s006.tif]
